# CAR T cells targeting tumor endothelial marker CLEC14A inhibit tumor growth

**DOI:** 10.1172/jci.insight.138808

**Published:** 2020-10-02

**Authors:** Xiaodong Zhuang, Federica Maione, Joseph Robinson, Michael Bentley, Baksho Kaul, Katharine Whitworth, Neeraj Jumbu, Elizabeth Jinks, Jonas Bystrom, Pietro Gabriele, Elisabetta Garibaldi, Elena Delmastro, Zsuzsanna Nagy, David Gilham, Enrico Giraudo, Roy Bicknell, Steven P. Lee

**Affiliations:** 1 Institute of Immunology and Immunotherapy, University of Birmingham, Birmingham, United Kingdom.; 2 Laboratory of Transgenic Mouse Models, Candiolo Cancer Institute, FPO-IRCCS, and Department of Science and Drug Technology, University of Torino, Torino, Italy.; 3 Radiation Therapy Laboratory, Institute for Cancer Research at Candiolo (IRCC), Torino, Italy.; 4 Institute of Inflammation and Ageing, University of Birmingham, Birmingham, United Kingdom.; 5 Clinical and Experimental Immunotherapy Group, University of Manchester, Manchester, United Kingdom.; 6 Institute of Cardiovascular Science, University of Birmingham, Birmingham, United Kingdom.

**Keywords:** Immunology, Oncology, Cancer immunotherapy, Immunotherapy, T cells

## Abstract

Engineering T cells to express chimeric antigen receptors (CARs) specific for antigens on hematological cancers has yielded remarkable clinical responses, but with solid tumors, benefit has been more limited. This may reflect lack of suitable target antigens, immune evasion mechanisms in malignant cells, and/or lack of T cell infiltration into tumors. An alternative approach, to circumvent these problems, is targeting the tumor vasculature rather than the malignant cells directly. CLEC14A is a glycoprotein selectively overexpressed on the vasculature of many solid human cancers and is, therefore, of considerable interest as a target antigen. Here, we generated CARs from 2 CLEC14A-specific antibodies and expressed them in T cells. In vitro studies demonstrated that, when exposed to their target antigen, these engineered T cells proliferate, release IFN-γ, and mediate cytotoxicity. Infusing CAR engineered T cells into healthy mice showed no signs of toxicity, yet these T cells targeted tumor tissue and significantly inhibited tumor growth in 3 mouse models of cancer (Rip-Tag2, mPDAC, and Lewis lung carcinoma). Reduced tumor burden also correlated with significant loss of CLEC14A expression and reduced vascular density within malignant tissues. These data suggest the tumor vasculature can be safely and effectively targeted with CLEC14A-specific CAR T cells, offering a potent and widely applicable therapy for cancer.

## Abstract 2

T cells expressing a chimeric antigen receptor specific for the tumor vascular marker CLEC14A inhibited tumor growth in three mouse cancer models.

## Introduction

Adoptive T cell therapy is clinically effective in some tumor settings (1, 2). However extending this approach to other cancers is limited by a paucity of defined tumor-specific antigens that can be safely targeted and, particularly for solid tumors, the inability of effector lymphocytes to access the malignant cells and/or function within the immunosuppressive tumor microenvironment. Therefore, rather than target the malignant cells directly, an alternative approach is to target the tumor vasculature upon which the cancer depends.

Endothelial cells that line the vasculature within tumors are subject to a different extracellular environment compared with endothelial cells in healthy tissue. For example, in tumors, they may be exposed to hypoxia, nutrient deprivation, more acidic conditions, and different mechanical forces such as reduced blood flow rate and increased mechanical compression (3). Accordingly, the tumor vasculature displays a different transcriptome compared with healthy tissues, with expression of tumor endothelial markers (TEMs) that could be targeted therapeutically.

Targeting the tumor vasculature offers several advantages over targeting malignant cells directly. First, endothelial cells are in direct contact with the circulation and therefore easily accessible to agents delivered i.v. Second, it is not necessary to kill the endothelial cells, since even temporary changes in their shape or function could lead to a blood clot and thereby destruction of surrounding tumor tissue. Third, shutting down 1 vessel will affect not only tumor cells in the immediate vicinity but also all tumor tissue downstream of this blood supply. Fourth, some TEMs are broadly expressed on the vasculature of different tumor types. Fifth, tumor vascular endothelial cells are more genetically stable than malignant cells (4) and, thus, less likely to generate antigen-loss immune escape variants.

The potency of T cell–based therapies for cancer probably reflects the cytotoxic nature of T-lymphocytes, as well as their ability to self-replicate and recruit other components of the cellular immune response. However, naturally occurring T cells specific for tumor antigens are often of low avidity due to immunological self-tolerance. Nevertheless, T cells can be genetically engineered to express high-affinity conventional or chimeric T cell receptors (5, 6). T cells engineered to express a chimeric antigen receptor (CAR) targeting CD19 have mediated remarkable clinical responses in leukemia and lymphoma patients (7). CARs usually comprise a monoclonal antibody–derived single-chain variable fragment (scFv; consisting of a heavy and light chain joined by a flexible linker) fused through a transmembrane domain to the cytoplasmic CD3ζ chain. More recently, constructs have incorporated additional cytoplasmic signaling domains from costimulatory molecules such as CD28 or 4-1BB to enhance CAR T cell survival in vivo (8). CARs therefore combine the specificity and affinity of a tumor-specific antibody with the potent effector functions of self-replicating T cells. Importantly, since CARs recognize native cell surface antigens independent of antigen processing, they are neither MHC restricted nor dependent on the antigen processing capacity of target cells. Therefore, they can be applied to all patients, regardless of HLA type, and recognize tumors with downregulated HLA expression.

Currently, most CAR therapy approaches are targeting antigens expressed by the malignant cells, but some are targeting TEMs such as vascular endothelial cell growth factor receptor 1 (VEGFR1) and VEGFR2 (9–11), prostate-specific membrane antigen (PSMA) (12), α_v_β_3_ integrin (13), TEM8 (14), or EIIIB, a splice variant of fibronectin (15). In all cases, T cells expressing CARs specific for the TEM mediated significant inhibition of tumor growth in mouse models, although mouse studies have also reported toxicity when targeting some of these markers (9, 16). To date, only a VEGFR2-specific CAR has been tested clinically; out of 24 patients treated, 1 patient had a partial response, but others were unresponsive (NCT01218867, https://clinicaltrials.gov/ct2/show/results/NCT01218867). Clinical trials of PSMA-specific CARs are ongoing.

Previously, we identified the glycoprotein CLEC14A as a TEM, highly expressed on the surface of vascular endothelial cells in many common human cancers but expressed at low or undetectable levels in healthy tissue (17). More recently, we have conducted an extensive analysis of CLEC14A protein and gene expression in healthy and diseased human and primate tissues, and we confirmed this protein to be a highly promising target, especially in renal cell cancer (18). CLEC14A expression can be induced under conditions of low shear stresses (17), such as occur in ill-formed vessels of tumor tissue, but other mechanisms may also contribute, including activin receptor–like kinase 1 signaling (19) and hypoxia (20). CLEC14A is a member of the type 14 family of calcium-dependent C-type lectins that includes endosialin/TEM1/CD248, thrombomodulin, and CD93 (21). This single-pass type I transmembrane protein (499 amino acids [aa] long) contains one C-type lectin–like domain (aa 33–173) and an epidermal growth factor–like domain (aa 245–287) in the extracellular region (UniProt). Human and mouse CLEC14A proteins show 67% aa sequence identity, with 78% and 79% identity within the C-type lectin and epidermal growth factor-like domains, respectively. It interacts with multimerin-2 within the extracellular matrix (22) and regulates VEGFR2- and VEGFR3-dependent signalling (23). CLEC14A mediates filipodia formation and endothelial migration (17, 24), plays a role in sprouting angiogenesis, and promotes tumor growth in mice (22).

In the present study, we explored targeting tumor vasculature using CLEC14A-specific CAR-expressing T cells. Having characterized the function of these cells in vitro, we then explored their efficacy and preliminary safety using 3 mouse tumor models.

## Results

### Generation and expression of CLEC14A-specific CARs.

**Figure 1:**
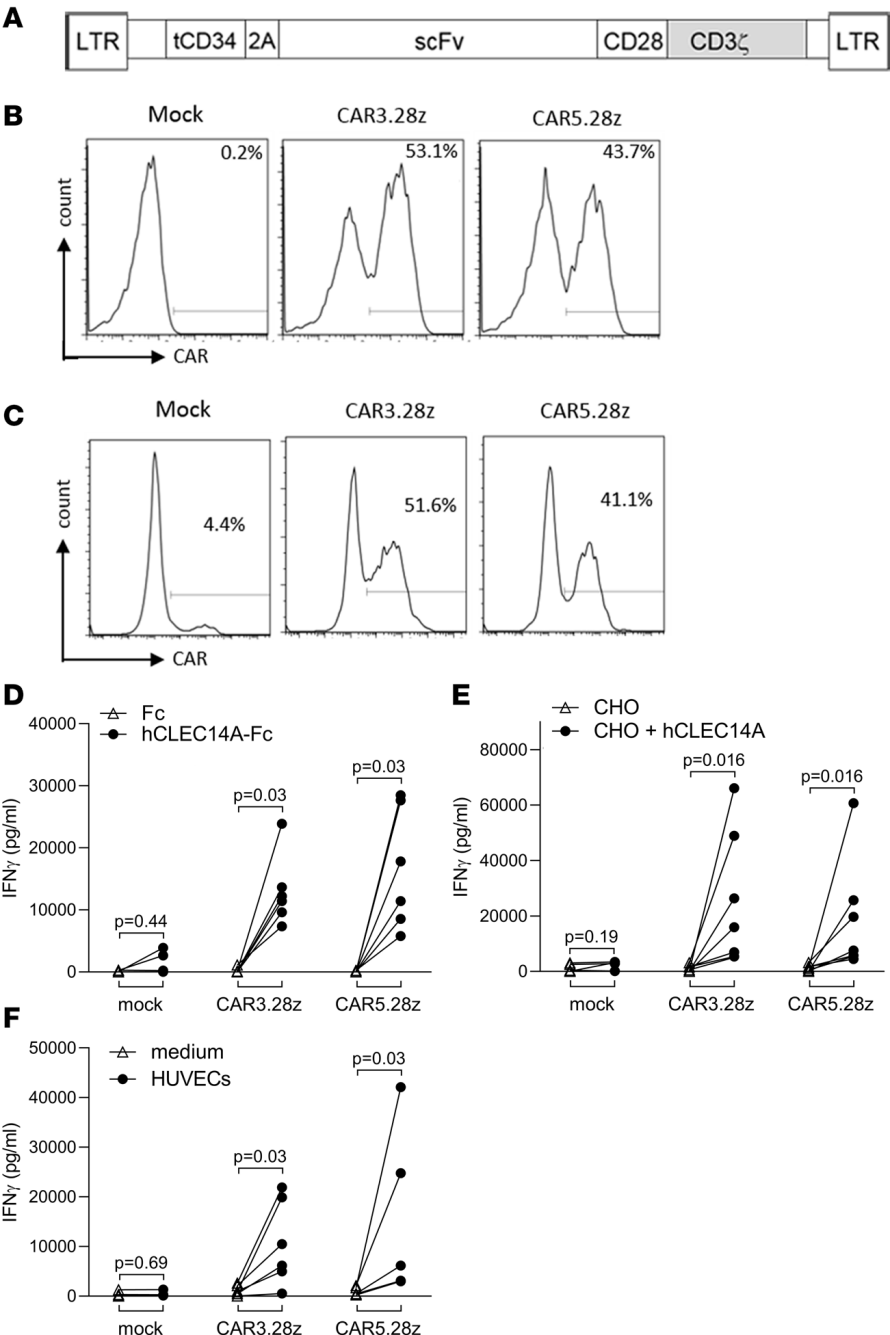
CLEC14A-specific CAR design, expression, and function. (**A**) Schematic representation of a recombinant retroviral vector encoding CLEC14A-specific CARs. Retroviral CAR vector (pMP71) coexpresses a truncated CD34 marker gene and an scFv fragment/CD3ζ chain chimeric receptor with a CD28 costimulatory domain. Expression is driven from the LTR promoter, and the 2A peptide linker ensures equimolar expression of both molecules. (**B**) Recombinant retroviral expression vectors encoding CARs CAR3.28z and CAR5.28z (with scFv fragments from CLEC14A-specific monoclonal antibodies CRT3 or CRT5, respectively) were used to transduce human T cells. CAR expression was detected by staining for the CD34 marker. Percentage values show proportion of cells stained for CD34 compared with mock-transduced T cells. (**C**) Transduced T cells stained for expression of CAR using CLEC14A-Fc (percentage values show specific binding of CLEC14A-Fc having subtracted background staining with Fc alone). (**D**–**F**) Human T cells engineered to express putative CLEC14A-specific CARs (or mock-transduced T cell controls) were tested using an ELISA for IFN-γ production for response to plate-bound recombinant CLEC14A-Fc (extracellular domain) fusion protein (or Fc alone) (**D**), CHO cells engineered to express full-length human CLEC14A (or CHO transduced with vector alone) (responder/stimulator [R/S] ratio = 6:1) (**E**), and HUVECs naturally expressing CLEC14A (or medium alone). (R/S ratio = 1:1) (**F**). In all cases, the different CAR T cell lines were diluted with mock T cells to equalize for transduction efficiency. Cells were stimulated for 18 hours before testing for IFN-γ production. Results of ELISAs show data from 6–7 repeat experiments, having subtracted background responses of T cells alone. All *P* values shown were calculated using a Wilcoxon matched-pairs signed rank test.

Monoclonal antibodies to CLEC14A that cross-react with human and mouse forms of the protein were previously generated by vaccinating mice with the extracellular domain of mouse CLEC14A. To confirm the specificity of 2 of these antibodies (CRT3 and CRT5), they were used to stain HUVECs following siRNA knockdown of human CLEC14A protein (note CLEC14A is naturally expressed in HUVECs under static culture conditions; ref. 17). Using flow cytometry, clear staining was detected on HUVECs treated with a noncomplementary siRNA duplex control, whereas staining of HUVECs treated with siRNA targeting CLEC14A was reduced to almost background levels (Supplemental Figure 1; supplemental material available online with this article; https://doi.org/10.1172/jci.insight.138808DS1). Using reverse transcription PCR (RT-PCR) and degenerate primer sets, a gene construct encoding an scFv was then generated for each of these antibodies and cloned into a retroviral vector to encode a second-generation CAR in which the scFv was linked to the human CD28 costimulatory domain and human CD3ζ chain (Figure 1A). In all constructs, the CAR gene was separated from a truncated human CD34 marker gene by a foot-and-mouth disease virus 2A peptide linker ensuring equimolar expression of both genes. In this way, CD34 expression acted as a marker for CAR expression.

Human T cells were then transduced with these retroviral constructs and analyzed by flow cytometry. As illustrated in Figure 1B, CD34 expression was readily detected in T cells transduced with vectors encoding CARs based on either of the 2 CLEC14A-specific antibodies. Using recombinant CLEC14A protein, it was also possible to stain directly for surface CAR expression (Figure 1C).

### In vitro functions of CLEC14A-specific CAR engineered T cells.

In vitro tests were used to assess the function of these engineered T cells. Using an ELISA to detect IFN-γ release, T cells expressing the CARs were diluted with mock T cells to equalize the proportion of transduced cells, and they were then compared for their ability to respond to human CLEC14A. The target antigen was expressed either as a recombinant Fc-fusion protein immobilized on a plate, overexpressed on the surface of engineered CHO cells, or naturally expressed at physiological levels on the surface of HUVECs grown under static culture conditions. As shown in Figure 1, D–F, in all cases, there was a specific response to CLEC14A above control targets. Note that these CAR T cells also produced the cytokines TNF-α and IL-2 in response to CLEC14A (Supplemental Figure 2).

**Figure 2:**
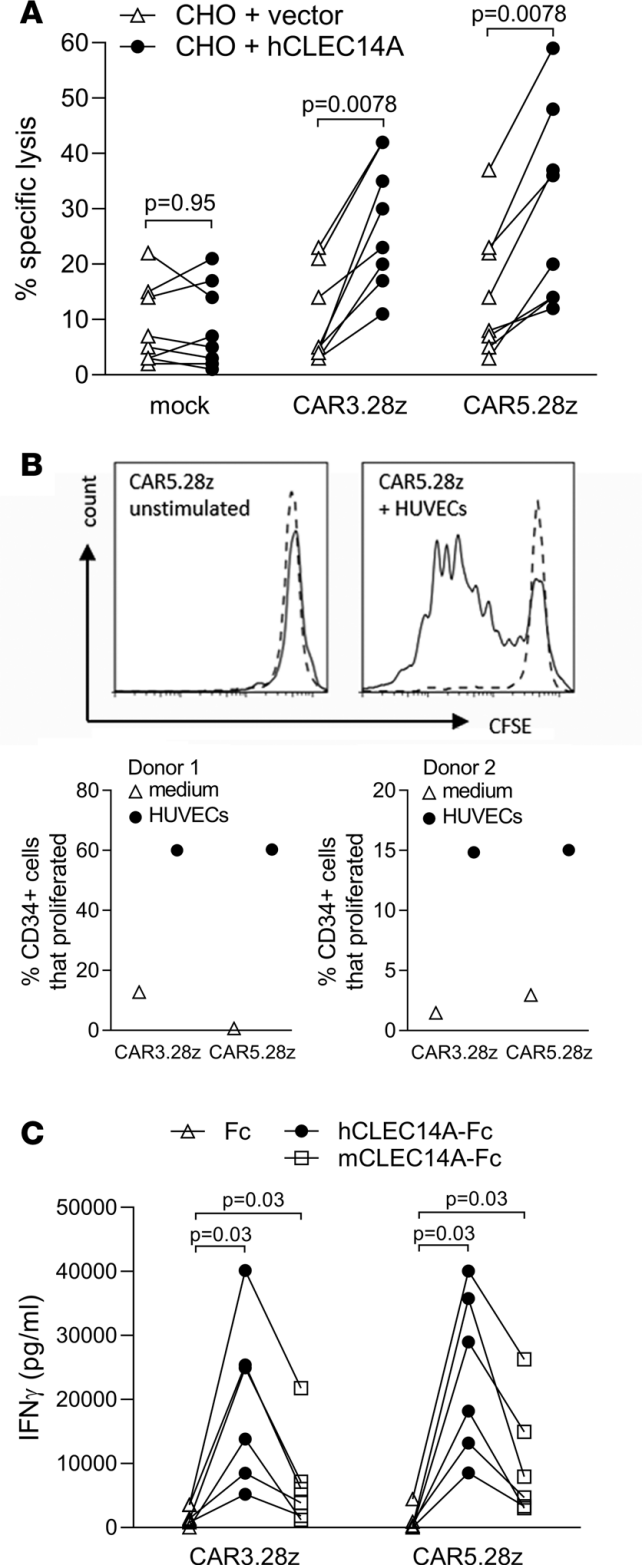
Further characterization of functional responses in CAR-transduced T cells. (**A**) Human T cells expressing CLEC14A-specific CARs (or mock T cell controls) were tested for cytotoxicity against CHO cells engineered to express full-length human CLEC14A (or control CHO cells transduced with vector alone). Results show data from 8 repeat experiments (effector/target ratio = 9:1). (**B**) Such T cells were also tested for proliferation, measured by CFSE staining of CD34^+^ T cells (solid line) and CD34^–^ T cells (dotted line) when cocultured with HUVECs or medium alone (unstimulated). Results show a histogram of T cells expressing CAR5.28z, and the 2 graphs below show data from 2 repeat experiments giving the percentage of CD34^+^ cells that proliferated for each of the CARs indicated (having subtracted the percentage of CD34^+^ T cells that proliferated in medium alone). (**C**) CLEC14A-specific CAR T cells (or mock T cell controls) were also tested for IFN-γ release in response to plate-bound recombinant human or mouse CLEC14A (both expressed as Fc-fusion proteins) or to Fc alone. Results show data from 6 repeat experiments. All *P* values shown were calculated using a Wilcoxon matched-pairs signed rank test.

Using a chromium release assay, we assessed the cytotoxic function of the CAR T cells. CHO cells expressing human CLEC14A (or CHO cells plus vector only control) were cocultured with CAR T cells or mock T cells. Again CAR T cell preparations were diluted with mock T cells to equalize for transduction efficiencies. Both CAR constructs tested mediated specific lysis of CLEC14A^+^ targets (Figure 2A).

CFSE labeling of CAR T cells demonstrated that they can also proliferate when cultured with HUVECs. This proliferation was induced only in CD34^+^ T cells and not in the nontransduced (CD34^–^) subset within the T cell preparation, indicating that it is in response to CLEC14A (Figure 2B).

Next, we sought to compare responses of our CAR T cells with human and mouse versions of CLEC14A. CAR T cell preparations were diluted with mock T cells to equalize for transduction efficiencies and cultured in wells precoated with recombinant CLEC14A-Fc fusion proteins (or Fc alone). Results shown in Figure 2C demonstrate that T cells expressing either CAR3.28z or CAR5.28z responded to mouse CLEC14A, albeit to a lesser degree than their response to human CLEC14A. Given this response to mouse CLEC14A, further studies on safety and antitumor effects were possible in vivo using mouse models.

### Expression profile of CLEC14A in mouse organs.

**Figure 3:**
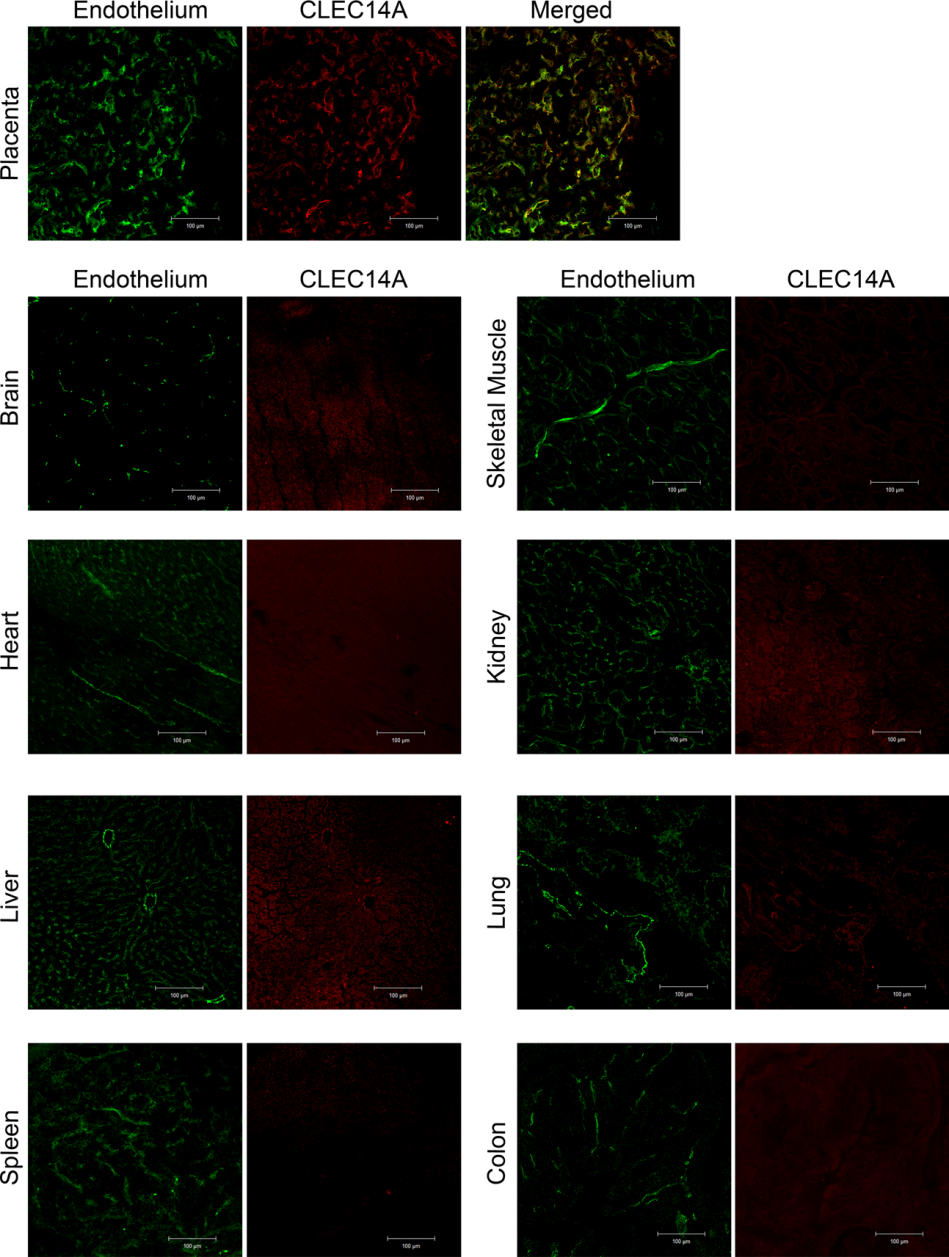
Immunofluorescence staining for CLEC14A in healthy mouse tissues. Confocal images of CLEC14A immunofluorescence staining of healthy mouse tissue. Green, MECA32 (kidney) or CD31 (all other tissues) staining for endothelial cells. Red, CLEC14A staining. Scale bars: 100 μm.

In preparation for in vivo studies, we first needed to determine the expression of CLEC14A in healthy mouse tissues to see if it mirrored that seen in human tissues. Therefore, we performed immunofluorescence staining for CLEC14A on frozen tissue sections from multiple organs collected from 3 healthy WT female adult C57BL/6J mice. Results demonstrated that expression does indeed mirror that seen in human tissue, with little or no CLEC14A detectable in adult mouse tissues. As in humans (data not shown), CLEC14A was detected in the placenta, a site of active angiogenesis, and this colocalized with the vasculature (Figure 3).

### Safety testing of CAR T cells in healthy mice.

Having shown that the mouse is an appropriate model for safety testing, C57BL/6J mice were conditioned with 4 Gy total body irradiation (TBI) to aid T cell engraftment and subsequently infused i.v. with 20 million T cells/mouse (where 20% of T cells expressed either CAR3.28z or CAR5.28z). As a control, other mice received 20 million mock-transduced T cells. Infused T cells were generated from a congenic (CD45.1) mouse strain and were, thus, distinguishable from host T cells. Mice were monitored carefully for toxicity over the next 6 weeks, and using serial blood samples, infused CAR T cells were tracked by CD34 and CD45.1 staining.

**Figure 4:**
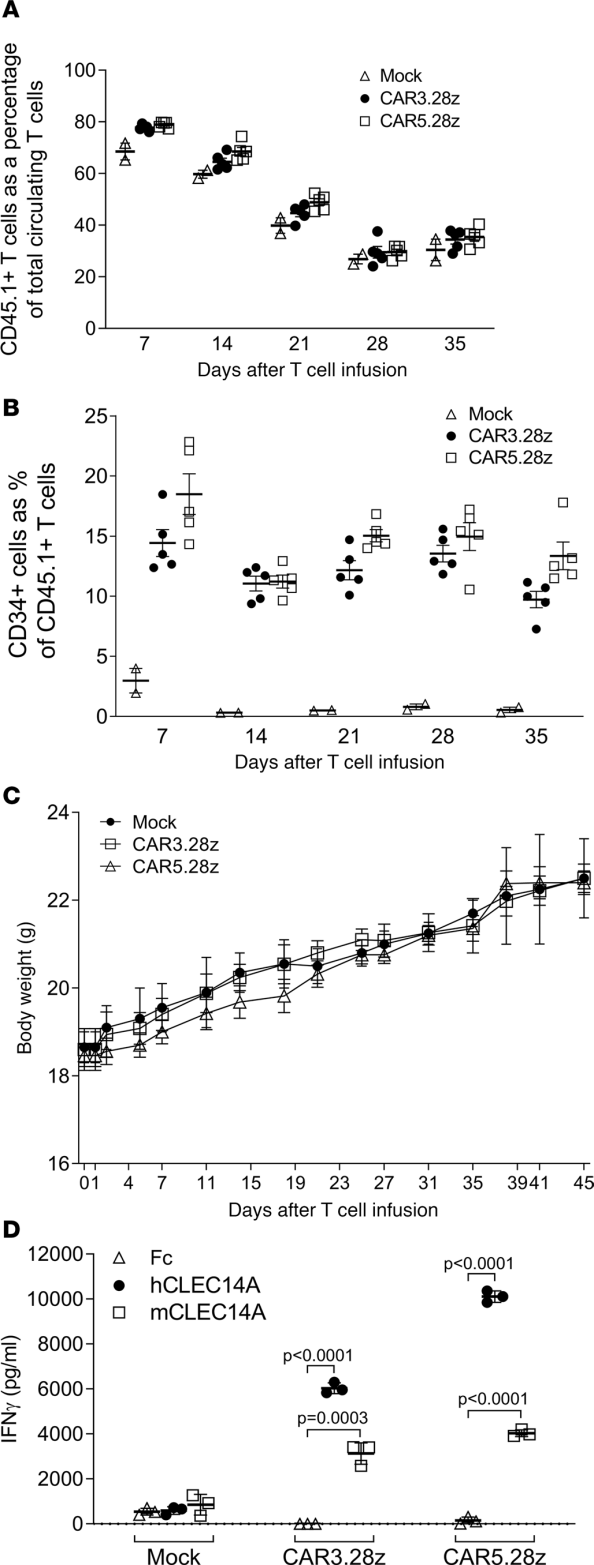
Toxicity testing of mouse T cells expressing CLEC14A-specific CARs in healthy mice. C57BL/6 mice were conditioned by irradiation and then infused with mouse T cells from a congenic (CD45.1^+^) strain. These T cells expressed CAR3.28z (*n* = 5), CAR5.28z (*n* = 5), or no CAR (mock; *n* = 2). (**A** and **B**) Serial blood samples were analyzed by flow cytometry to determine the percentage of infused T cells in the circulating T cell pool (**A**) and the percentage of CAR-expressing (CD34^+^) T cells in the infused T cell population (**B**). (**C**) The animals showed no signs of toxicity, as illustrated by their consistent increase in body weight. Results in **A**–**C** show the mean ± SEM. (**D**) Ex vivo analysis of CAR-expressing T cells (or mock T cells) recovered from the spleens of animals at the end of the experiment tested by ELISA for their ability to release IFN-γ when exposed to recombinant human or mouse CLEC14A protein. Results in **D** show the mean of triplicate cultures (±SD), with *P* values calculated using an unpaired *t* test, and are representative of 2 repeat experiments. All *t* tests were 2 tailed.

**Figure 5:**
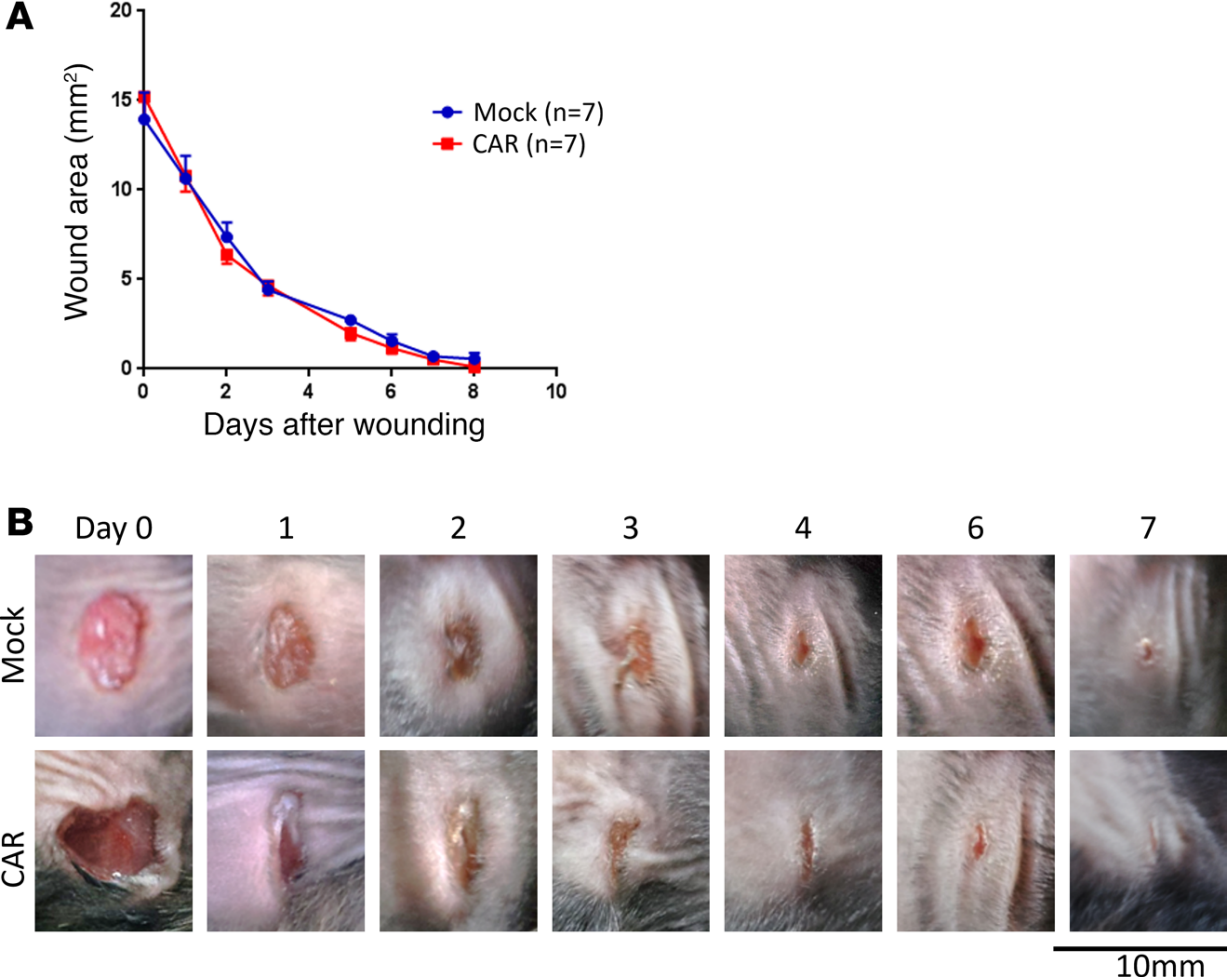
CLEC14A-specific CAR T cells do not affect wound healing. C57BL/6 mice were injected s.c. on the flank with 1 × 10^6^ LLC cells and, 4 days later, injected with CLEC14A-specific CAR T cells (*n* = 7) or mock-transduced cells (*n* = 7). The next day, skin wounds were administered to the opposite flank of the mouse. (**A**) The size of the wound area was recorded over time (*n* = 7 mice per group; data shown are mean ± SEM). (**B**) Representative images of the wounds from mock- and CAR-treated mice at the time points indicated.

As shown in Figure 4A, the infused (CD45.1^+^) T cells were detectable throughout this period, initially representing the majority of T cells within the circulation of the mice. Their relative abundance gradually decreased with time, probably due to cell death and/or recovery of the host’s endogenous (CD45.1^–^) T cell population after conditioning. Nevertheless, the proportion of infused T cells that expressed the CARs (CD34^+^) remained relatively consistent throughout this period (Figure 4B), suggesting that the infused T cells were not responding to target antigen in healthy tissues. Despite the presence of CAR T cells, the mice showed no ill effects, as illustrated by a consistent gain in body weight in both CAR- and mock-treated animals (Figure 4C). On day 45, mice were sacrificed, and vital organs were collected and H&E stained for analysis by an experienced mouse histopathologist; no signs of pathology were observed (Supplemental Figure 3). Importantly, at the end of the experiment, ex vivo analysis of CD34^+^ CAR T cells sorted from the spleens of these animals demonstrated that the CAR T cells had retained functional activity, producing IFN-γ in response to both human and mouse recombinant CLEC14A proteins (Figure 4D). When 10 million T cells expressing a CLEC14A-specific CAR were injected into a knockin mouse expressing the human rather than mouse CLEC14A extracellular domain, we again observed no toxicity (Supplemental Figure 4). The CAR T cells did not affect wound healing in WT mice (Figure 5).

### Antitumor responses with CLEC14A-specific CAR engineered T cells.

To determine the ability of the engineered T cells to inhibit tumor growth, we used 3 mouse tumor models. In vitro studies had shown little difference between CAR3.28z and CAR5.28z, so we began analyzing antitumor effects in vivo by focusing on CAR5.28z-expressing T cells. T cells from C57BL/6J mice were engineered to express CAR5.28z (or mock-transduced) and injected i.v. into 12-week-old Rip-Tag2 mice. These mice develop hyperplastic islets at 3–4 weeks of age, and by 10 weeks, small encapsulated adenomas emerge. By 12–13 weeks, these adenomas become much larger, and death normally occurs at approximately 14 weeks.

To perform a regression trial, CAR-treated Rip-Tag2 mice (*n* = 12 per group) received a total of 15 million cells, of which 2.4 million expressed CAR5.28z. As a control, another 12 Rip-Tag2 mice were injected with 15 million T cells that lacked the CAR (i.e., mock-transduced). To aid T cell engraftment, all animals were irradiated (4 Gy) 24 hours before T cell infusion. As previously described (25–27), all mock-treated animals were culled at 14 weeks of age due to tumor growth, and pancreatic dysfunction and tumor size was measured. CAR-treated animals were analyzed at 16 weeks of age, allowing for the standard 4-week time period of treatment used for the regression trial (25).

**Figure 6:**
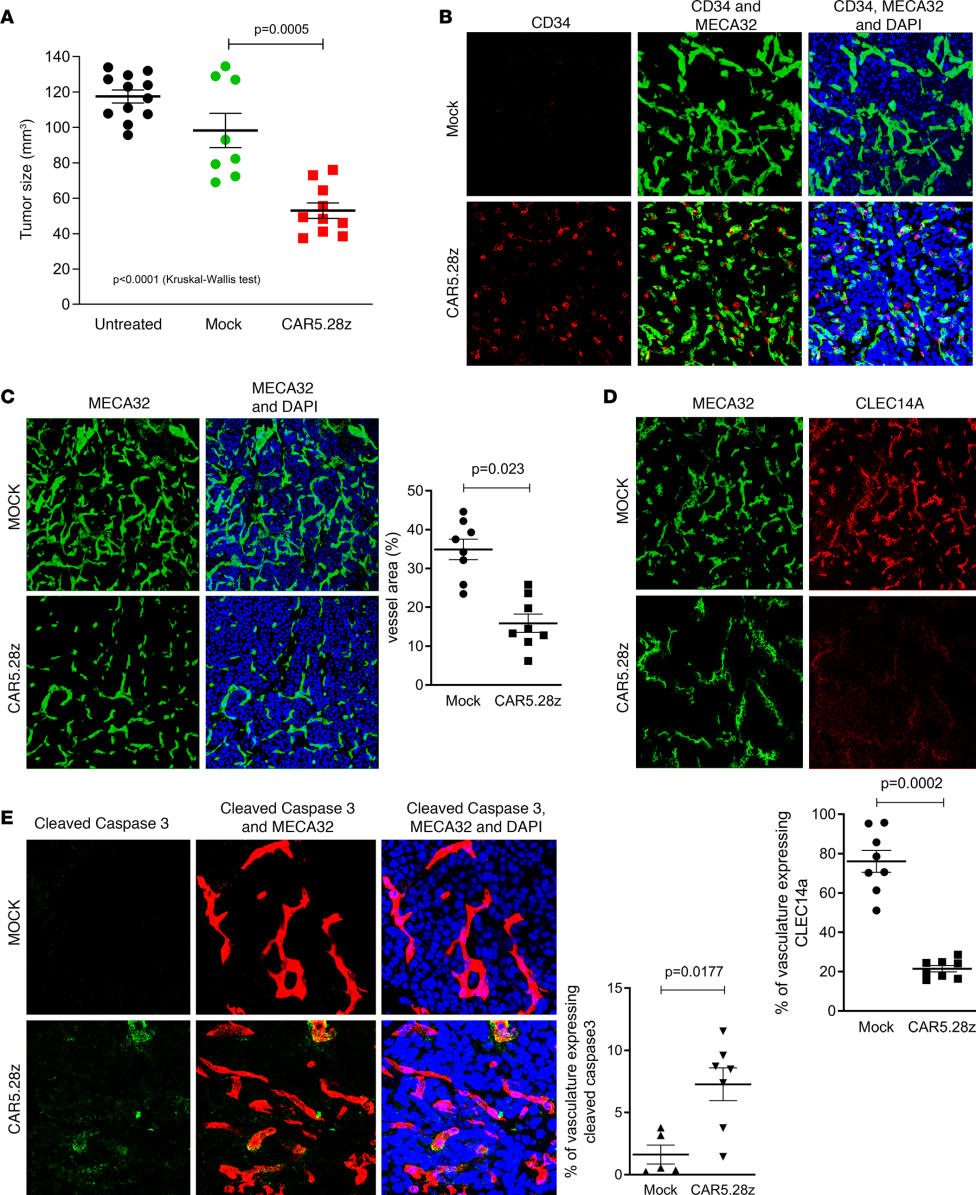
Antitumor responses in Rip-Tag2 mice. Rip-Tag2 mice at 12 weeks of age were conditioned with 4 Gy total body irradiation and then infused with CAR5.28z-expressing mouse T cells (*n* = 12) or mock-transduced mouse T cells (*n* = 12). (**A**) Tumor size was measured at 14 or 16 weeks of age for mock- and CAR-treated animals, respectively. Results show tumor size for individual mice that survived to the end of the experiment, along with the mean ± SEM. Tumor sizes in 12 untreated (nonirradiated) mice measured at 14 weeks of age are included as a control. (**B**) CAR-transduced (CD34^+^) T cells were detectable by immunofluorescent imaging in CAR-treated RIP-Tag2 tumors 4 weeks after injection (red, CD34; green, MECA32 endothelial marker; blue, DAPI stain). Staining of tumor tissue from mock-treated animals is included as a control. **C**–**E** show data from immunofluorescent imaging of CAR- and mock-treated tumor tissue, with representative images of staining plus a scatterplot of results from individual mice. (**C**) Data on vascular density (green, MECA32 endothelial marker; blue, DAPI). (**D**) CLEC14A expression in vessels (green, MECA32; red, CLEC14A). (**E**) Proportion of apoptotic vessels (green, cleaved caspase-3; red, MECA32; blue, DAPI). All scatterplots show mean ± SEM. All *P* values shown were calculated using a 2-tailed Mann-Whitney *U* test (except where indicated in **A**, where the 3 test groups were compared using a Kruskal-Wallis test).

As shown in Figure 6A, tumor size in CAR-treated mice was significantly reduced compared with mock-treated animals, even though CAR-treated tumors were allowed to grow for a further 2 weeks. Four of 12 mock-treated animals died before 14 weeks of age and were excluded from the analysis of tumor size. In contrast, all 12 CAR-treated animals survived to 14 weeks, and remarkably, all but 2 survived to 16 weeks of age. These data suggest that CAR T cell treatment is able to regress pancreatic tumor and, at the same time, prolong the survival of Rip-Tag2 mice. Of note, the mean tumor size in mock-treated animals was lower than in untreated (nonirradiated) mice, implying irradiation of mock-treated animals may have partially affected tumor growth, although this difference was not significant (*P* = 0.15, Mann-Whitney *U* test). Staining for the CD34 marker in tumor tissue recovered from mice at the end of this experiment demonstrated that CAR-expressing T cells were present at the tumor site (Figure 6B). There was also a statistically significant decrease in both the total number of vessels and the proportion of CLEC14A-expressing vessels within tumors of CAR-treated mice compared with the mock-treated controls (Figure 6, C and D). Cleaved caspase-3 expression in these tumors indicated that CAR treatment was inducing apoptosis in the tumor vasculature (Figure 6E).

**Figure 7:**
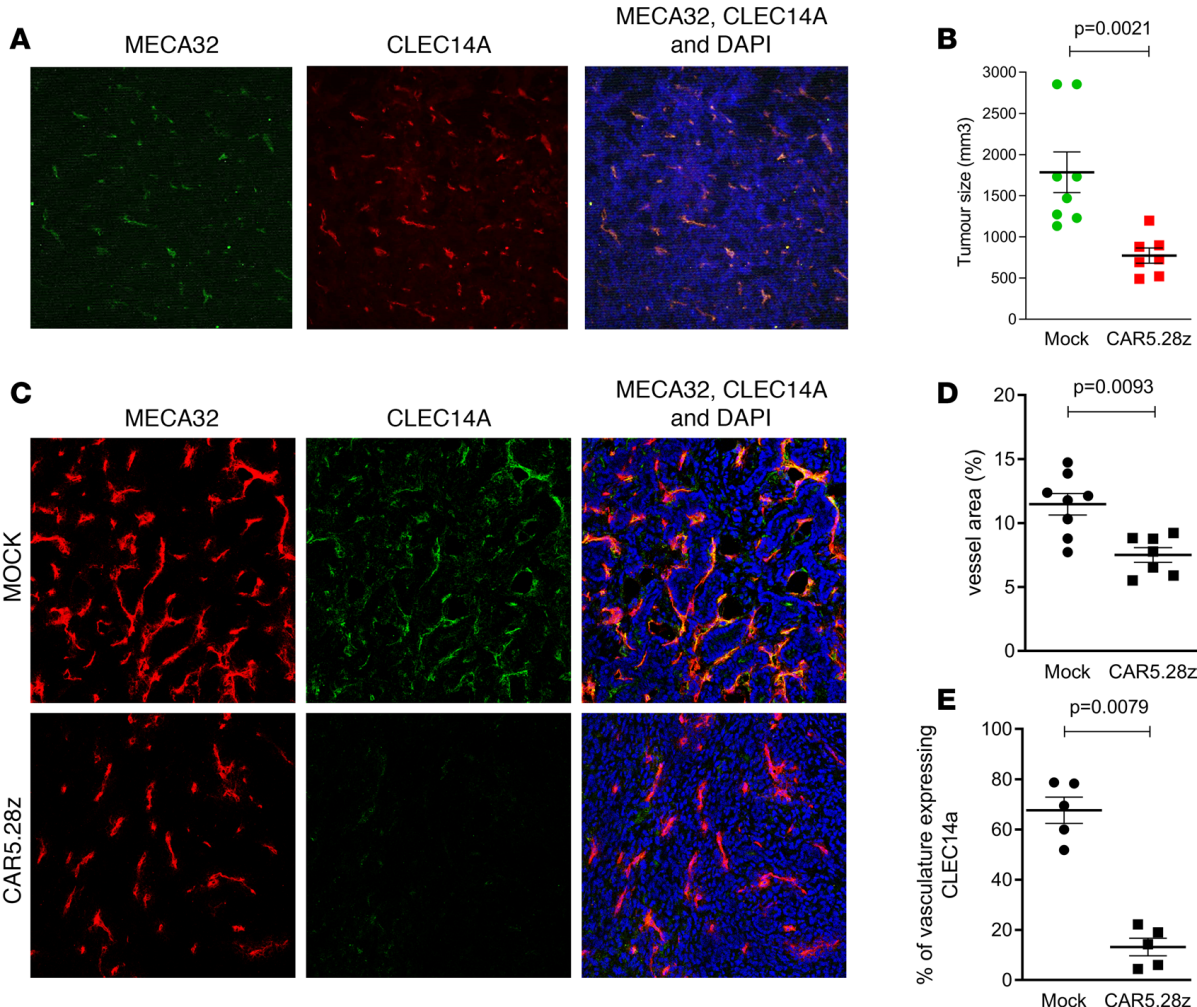
Antitumor responses in PDAC mice. (**A**) CLEC14A expression in PDAC tumor tissue (green, MECA32 endothelial marker; red, CLEC14A). FVB/n mice were injected into the pancreas with PDAC tumor cells and then conditioned 1 week later with cyclophosphamide. The following day, mice were infused with CAR5.28z-expressing mouse T cells (*n* = 7) or mock-transduced mouse T cells (*n* = 8). Three weeks later, mice were culled and tumor sizes were measured. (**B**) Results show tumor size for individual mice. (**C**–**E**) Representative images of immunofluorescent staining of CAR- and mock-treated tumor tissue (red, MECA32 endothelial marker; green, CLEC14A; blue, DAPI; **C**) to determine vascular density (**D**) and density of CLEC14A^+^ vessels (**E**). All scatterplots show data from individual mice, with mean ± SEM. All *P* values shown were calculated using a 2-tailed Mann-Whitney *U* test.

A second pancreatic tumor model, murine pancreatic ductal adenocarcinoma (mPDAC), also expresses high levels of CLEC14A in the tumor vasculature (Figure 7A). PDAC tumor cells were injected into the pancreas of FVB/n mice, and 1 week later, mice were conditioned using cyclophosphamide to aid T cell engraftment. The following day, CAR5.28z- or mock-transduced syngeneic T cells were injected into the tail vein, and tumor size was measured 3 weeks later. As shown in Figure 7B, there was a significant inhibition of tumor growth in CAR-treated mice, and histological examination of tumor tissue demonstrated that there was also a significant reduction in the vasculature of CAR-treated tumors, with loss of CLEC14A-expressing vessels (Figure 7, C–E).

**Figure 8:**
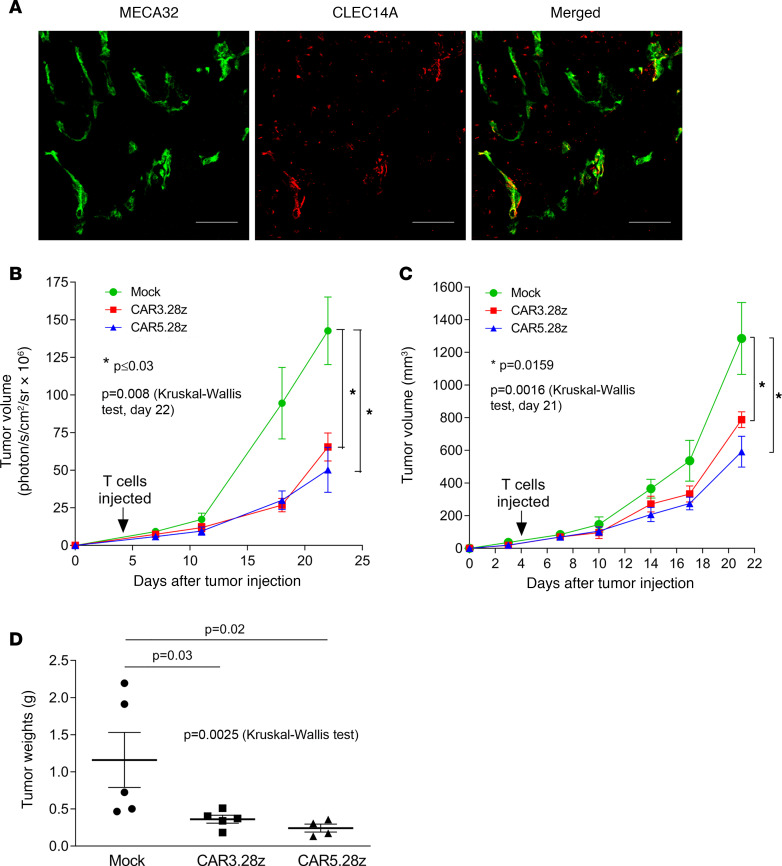
Antitumor responses in the Lewis lung carcinoma mouse model. (**A**) CLEC14A expression in Lewis lung carcinoma tissue (green, MECA32 endothelial marker; red, CLEC14A). Scale bar: 50 μm. C57BL/6 mice were s.c. injected with LLC cells and 4 days later infused with mouse T cells expressing CAR3.28z (*n* = 5), CAR5.28z (*n* = 4), or mock transduced (*n* = 5). (**B** and **C**) Tumor size was measured at the time points indicated using bioluminescence (**B**) or calipers (**C**). (**D**) At the end of the experiment (day 22 after tumor inoculation), tumors were resected and weighed. All scatterplots show data from individual mice with mean ± SEM. All graphs show mean ± SEM. All *P* values shown were calculated using a 2-tailed Mann-Whitney *U* test, except when comparing all 3 groups, where a Kruskal-Wallis test was used (as indicated).

Further studies explored the antitumor effects of CAR5.28z using the syngeneic LLC mouse model. Here, we tested CAR3.28z to determine if this other construct was also capable of inhibiting tumor growth. When injected s.c. on the flank of a C57BL/6J mouse, LLC cells grow rapidly, forming large tumors within 3–4 weeks. Previous studies have demonstrated that CLEC14A is upregulated on vessels in this tumor and promotes tumor growth (22). Staining LLC for CLEC14A protein confirmed expression on some vessels within these tumors (Figure 8A). Four days after tumor inoculation, mice were injected i.v. with syngeneic T cells expressing either CAR3.28z or CAR5.28z (or mock transduced). Over the next 18 days, there was a statistically significant inhibition of tumor growth in CAR-treated mice compared with mice treated with mock-transduced T cells. This was apparent from bioluminescent imaging of the luciferase-expressing tumor cells, caliper measurements of the same tumors, and tumor mass at the end of the experiment (Figure 8, B–D).

Importantly, no CAR T cell–mediated toxicity was apparent in any of these tumor mouse models, supporting the data obtained in our studies with healthy mice.

## Discussion

Tumor vasculature not only supplies oxygen and nutrients to aid tumor growth, but it can also provide factors that maintain self-renewing cancer stem cells (28) and release growth signals that promote malignant cell proliferation (29). Furthermore, tumor endothelium may contribute to immune escape through expression of receptors that inhibit T cell activation (e.g., PD-L1, PD-L2) (30) and FasL expression, which preferentially kills CD8^+^ T cells but not Tregs (31). Furthermore, activated endothelin B receptor on tumor endothelium can inhibit T cell recruitment (32), whereas upregulation of common lymphatic and vasculature endothelial receptor-1 (CLEVER1) may selectively enhance Treg recruitment (33). Consequently, tumor vasculature plays an important role in tumor growth and survival, and so targeting it for destruction should significantly impact disease.

Therefore, the present study explored the efficacy and preliminary safety of targeting a TEM CLEC14A. CLEC14A plays a role in angiogenesis (17, 22), so targeting it should not only eliminate existing tumor vasculature, but it should also inhibit formation of new blood vessels in tumor tissue. Previous work with a CLEC14A-specific antibody demonstrated antiangiogenic and antitumor effects in the LLC mouse model by blocking the interaction between CLEC14A and multimerin-2 (22). However, given the potent cytotoxic and immunoregulatory activity of self-replicating T cells, we reasoned that CARs could be more effective than blocking antibodies, since they act as vascular disrupting agents with cytotoxic rather than cytostatic functions. Our CLEC14A-specific CAR T cells were capable of IFN-γ release, cytotoxicity, and proliferation in response to their target antigen, indicating their antitumor potential. We incorporated the CD28 costimulatory domain into our CAR constructs, as previous studies have demonstrated that this enhances the function of these cells (8). Alternative or additional costimulatory domains (e.g., from 4-1BB) may further enhance the antitumor efficacy of CAR T cells by increasing their persistence and altering their metabolic profile (34).

Encouragingly, when injected into healthy mice, we demonstrated persistence of functional CLEC14A-specific CAR T cells without toxicity. This is consistent with our findings that CLEC14A is absent or expressed at low levels in healthy mouse tissues. Importantly, the same expression pattern is found in humans (17, 18). In vitro, our CAR T cells responded more strongly to human CLEC14A than the equivalent mouse protein (Figure 2C and Figure 4D). Therefore, we also tested for toxicity using healthy mice expressing the human CLEC14A extracellular domain, but again, no toxicity was observed. There was also no effect on wound healing rates in tumor-bearing WT mice. Not only did the CAR T cells appear safe, but following a single injection, they mediated significant inhibition of tumor growth in 3 different tumor models, including spontaneous and orthotopic tumors and 2 different mouse strains, with evidence of T cell targeting and destruction of the CLEC14A-expressing tumor vasculature. Before clinical studies can commence, further testing of the CARs when expressed in human T cells is required, but these results strongly support the therapeutic potential of this approach.

Although the CLEC14A-specific CARs inhibited tumor growth, some tumor tissue remained, supported by a reduced vasculature that largely did not express the target antigen (Figure 6, C and D). Therefore, to improve the efficacy of CLEC14A-specific CAR-based therapy for solid tumor vasculature, future studies could explore combining this approach with CARs targeting other TEMs to increase the proportion of tumor vessels that can be targeted. It may also be possible to further engineer the CAR T cells to improve their function in a hostile immunosuppressive tumor microenvironment. For example, coexpression of cytokines IL-12 (35) or IL-15 (10) by other CAR T cells targeting the vasculature increased their antitumor efficacy. Engineering expression of dominant-negative receptors may also assist in the avoidance of the immunosuppressive effects of cytokines such as TGF-β at the tumor site (36). Nevertheless, as with any vascular-targeting approach, there is the possibility that a viable rim of tumor cells will survive, supported by vasculature present in surrounding healthy tissue. Thus, combining CARs that target the vasculature with approaches that target the malignant cells directly is likely to be most effective. Proof of principle has already been reported in a mouse tumor model that demonstrated a synergistic effect when CAR T cells targeting VEGFR2 were combined with CAR T cells targeting an antigen on the malignant cells (37). In this case, destroying the tumor vasculature may result in selection of “normal” vessels that more effectively support entry of tumor antigen–specific T cells into malignant tissue. CAR T cell–mediated targeting of tumor vasculature could be combined with chemotherapy, since the selected normal vessels could increase drug delivery to the tumor and the CAR T cells could eliminate poorly perfused regions of tumor that are inaccessible or resistant to chemotherapeutic agents. Selected normal vessels will also increase oxygenation of tumor tissue, which is important for effective radiotherapy. Indeed many studies have shown that other vascular disruption agents can be combined with chemotherapy or radiotherapy, leading to a synergistic antitumor effect (reviewed in ref. 38). If vascular disruption leads to areas of hypoxia, this might inhibit antitumor immunity and increase invasiveness of malignant cells. In this case, combining CAR therapy with hypoxia-selective bioreductive drugs may also be beneficial (39).

CAR T cells targeting the vasculature will disrupt the tumor microenvironment that could lead to release of tumor antigens. These T cells will also produce cytokines that may recruit other components of the immune system. In this way, CAR T cells targeting the tumor vasculature may promote epitope spreading where the host subsequently mounts an immune response to additional tumor-associated antigens (40). This could enhance antitumor efficacy by inducing direct targeting of the malignant cells, especially if combined with immune checkpoint inhibitors such as anti–PD-1 antibodies that can activate therapeutic host antitumor immunity and may prevent PD-1–mediated inactivation of the CAR T cells (41).

As mentioned above, the potency of some CAR T cell therapies has led to toxicity in some patients, either from cytokine release syndrome or on-target, off-tumor reactivity. We have found expression of CLEC14A to be substantially lower in healthy human tissue, but appropriate measures should be employed to reduce the risk of toxicity in clinical studies. Suicide gene strategies could terminate a toxic response, should it occur, and 2 such systems have already proven clinically effective (42, 43), although eliminating the engineered cells may also prevent any therapeutic effect. Reducing tumor burden before T cell infusion may reduce the risk of cytokine release syndrome, and use of the IL-6–blocking antibody tocilizumab can be an effective treatment, should this syndrome arise (7). To prevent off-target effects with CARs, several strategies have been proposed, including use of combinatorial receptor approaches, where the presence of 2 target antigens is required to trigger the T cell, thus permitting selective targeting of tumor cells, if they uniquely express both antigens (44). Finally, limiting expression of the CAR either through engineering T cells with RNA leading to transient receptor expression (45) or using a drug-inducible on-switch to regulate CAR expression levels (46) should allow for greater safety in initial clinical studies.

Given the widespread expression of CLEC14A in human solid tumors and the non-MHC restricted nature of CAR-expressing T cells, CLEC14A-specific CAR T cells could potentially treat a wide range of cancer patients. Furthermore, given the antitumor efficacy of these CAR T cells in mouse tumor models, clinical studies are warranted to further explore their therapeutic potential, including their use in combination therapies.

## Methods

### Generation of CAR constructs

Hybridomas expressing CLEC14A-specific monoclonal antibodies that cross-react with human and mouse forms of the protein were obtained as described (22). Gene constructs encoding an scFv were then isolated from each of the mouse hybridomas by RT-PCR using degenerate primer sets designed to amplify all mouse V-gene families (47). scFv genes were then subcloned into the previously described CAR vector pMP71.tCD34.2A.CD19.IEVζ (48) as a ClaI/NotI fragment, replacing the CD19-specific scFv. This vector was originally constructed using the MP71 retroviral expression plasmid (a gift from C. Baum, Hannover Medical School, Hannover, Germany) and coexpressed a truncated CD34 marker (49).

### Transduction of human and mouse T cells

To generate recombinant retrovirus for transducing human T cells, Phoenix amphotropic packaging cells were transfected with an MP71 retroviral vector and pCL ampho (Imgenex) using FuGENE HD (Roche) according to the manufacturer’s instructions. Recombinant retrovirus for transducing mouse T cells was generated in the same way but using Phoenix ecotropic packaging cells and pCL-Eco. Human PBMCs were isolated from heparinized blood by density gradient centrifugation (800*g* for 30 minutes at 20°C) on lymphoprep (Axis Shield). PBMCs were preactivated for 48 hours using anti-CD3 antibody (30 ng/mL; OKT3, eBioscience), anti-CD28 antibody (30 ng/mL; 37407, R&D Systems), and IL-2 (300 U/mL; Chiron) in RPMI1640 (MilliporeSigma) containing 10% FBS (PAA), 2 mM l-glutamine (Gibco, Thermo Fisher Scientific), 100 IU/mL penicillin (Gibco, Thermo Fisher Scientific), and 100 pg/mL streptomycin (Gibco, Thermo Fisher Scientific) (standard medium) plus 1% human AB serum (TCS Biosciences). Transduction of mouse T cells was conducted using mouse splenocytes preactivated for 48 hours with concanavalin A (2 μg/mL; MilliporeSigma) and mouse IL-7 (1 ng/mL; eBioscience) in standard medium. Preactivated human and mouse T cells were subsequently transduced (or mock transduced with conditioned supernatant from nontransfected phoenix cells) by spinfection in retronectin-coated (Takara) plates according to the manufacturer’s instructions. Human T cells were then cultured in standard medium plus 1% human AB serum (TCS Bioscience) with IL-2 (100 U/mL). After spinfection, mouse T cells were cultured for 24 hours in standard medium with IL-2 (100 U/mL) and were then purified using lymphoprep. Cryopreservation of mouse T cells was conducted using ice-cold RPMI1640 containing 50% FBS and 10% dimethyl sulfoxide (MilliporeSigma) and a Mr. Frosty cell-freezing device (Nalgene). Cells were then stored over liquid nitrogen. After thawing, viability and recovery of mouse T cells was generally above 50% and 70%, respectively. Where indicated, transduced cells were sorted by immunomagnetic selection using anti-CD34 microbeads (Miltenyi Biotec) according to the manufacturer’s instructions.

### Cell lines and recombinant proteins

Phoenix A and E (National Gene Vector Biorepository, Indiana University, Indianapolis, Indiana, USA), CHO, and Lewis lung carcinoma (LLC) (American Type Culture Collection, CRL-1642) cells were maintained in DMEM (MilliporeSigma) containing 10% FBS (PAA), 2 mM l-glutamine (Gibco, Thermo Fisher Scientific), 100 IU/mL penicillin (Gibco, Thermo Fisher Scientific), and 100 pg/mL streptomycin (Gibco). mPDAC (provided by Doug Hanahan and Ksenya Shchors, ISREC, EPFL, Lausanne, Switzerland) were isolated from tumor-bearing *p48*^cre^, *Kras^LSL_G12D^*, *p53^R172H/+^*, and *Ink4a/Arf*^fl/+^ mice and were cultured as previously described (50). CHO cells had been transduced with the pWPI vector (Addgene) expressing full-length human CLEC14A (or vector alone). HUVECs were isolated and cultured as described (17). Cell lines were screened for mycoplasma using MycoAlert detection kit (Lonza). Human and murine CLEC14A proteins (extracellular domains) with a human Fc tag were expressed in HEK293T cells and purified on protein A columns as described (22).

### siRNA knockdown of CLEC14A

Transfection with siRNA was performed as described (51) using the following siRNA duplexes: D1-GAACAAGACAATTCAGTAA and D2-CAATCAGGGTCGACGAGAA (EuroGentec).

### Flow cytometry

HUVECs were trypsinized and stained with CLEC14A-specific mouse monoclonal antibodies described above (10 μg/mL) or IgG1 isotype control (Dako) in 5% normal goat serum/PBS. Cells were washed, and bound antibodies were detected by incubating with R. phycoerythrin–conjugated (R-PE–conjugated) goat anti-mouse antibody (STAR132PE, Serotec). Dead cells were identified using propidium iodide. Human T cells were washed with PBS and stained with Live/Dead Fixable Violet Dead Cell Stain Kit (Invitrogen). Cells were then washed with flow buffer (0.5% w/v BSA + 2 mM EDTA in PBS; pH 7.2) and stained with anti–human CD4 (PE-conjugated, 555347), anti–human CD8 (FITC-conjugated, 555634) (both from BD Pharmingen), and anti–human CD34 (PE-Cy5; 343506, BioLegend). Heparinized mouse tail bleeds were treated with BD Pharm lyse (Becton Dickinson) before staining T cells as described above but using anti–mouse CD4–FITC (catalog 553046), –CD8-PE (catalog 553032), and –CD45.1–PE-Cy7 (catalog 560578) (BD Pharmingen). Alternatively, CAR expression was detected on human T cells directly by firstly blocking cells with human Fc fragment (10 μg/mL) and then incubating them with 10 μg/mL recombinant human CLEC14A-Fc fusion protein (or Fc control) followed by sheep anti-CLEC14A polyclonal antibody (AF4968, R&D Systems, 10 μg/mL). Finally, cells were stained with FITC-conjugated rabbit anti-sheep antibody (818611, Invitrogen). All incubations were conducted for 1 hour on ice. Cells were analyzed using a BD LSRII flow cytometer and FlowJo software (Tree Star Inc.).

TNF-α and IL-2 produced by T cells were detected by intracellular cytokine staining. First, T cells were incubated with CHO expressing CLEC14A (or vector alone) in standard medium, and after 90 minutes of incubation, Brefeldin A (MilliporeSigma) was added at a final concentration of 10 μg/mL. The cells were cultured for a further 16.5 hours and then stained with Live/Dead Fixable Violet Dead Cell Stain Kit, followed by anti–human CD4–ECD (6604727, Beckman Coulter), anti–human CD8-AmCyan (catalog 339188), and anti–human CD34–PECy5 (catalog 555823) (both from BD Biosciences). The cells were washed in PBS, fixed in 4% paraformaldehyde, treated with PBS containing 0.5% saponin, and stained with anti–TNF-α–PECy7 (25-7349-82, eBioscience) or anti–IL-2–PE (559334, BD Pharmingen). Cells were then washed in PBS and analyzed using a BD LSRII flow cytometer. Fluorescence minus one controls were used to define gates when analyzing data with FlowJo software.

### CFSE labeling

T cells were washed twice with PBS and incubated with 2.5 μM CFSE for 10 minutes at 37°C. The labeling reaction was quenched by adding RPMI1640 containing 10% FBS. Cells were washed, resuspended in standard medium plus 1% human AB serum and IL-2 (10 IU/mL) at 1.5 × 10^6^ cells/mL, and added to wells containing HUVECs to give a T cell/HUVEC ratio of 10:1. After 5 days of incubation at 37^o^C/5%CO_2_, cells were analyzed by flow cytometry as described above using anti–human CD34 (PE-Cy5).

### IFN-γ release assay

Stimulator cells (2.5 × 10^4^/well) were cocultured in triplicate with CD34^+^ CAR T cells at responder/stimulator ratios indicated. Alternatively 2 × 10^4^ CD34^+^ CAR T cells were incubated in wells precoated with recombinant protein (1 μg/mL). Cells were incubated at 37°C/5% CO_2_ in 100 μL/well of RPMI supplemented with 10% FBS and IL-2 (25 U/mL). After 18 hours, culture supernatant was tested for secreted IFN-γ using an ELISA (Pierce Endogen) according to the manufacturer’s instructions.

### Cytotoxicity assays

Chromium release assays have been described previously (52). They were set up at an effector/target ratio of 9:1 (1250 targets/well) and harvested after 7.5 hours.

### Tissue preparation and immunohistology

Fresh tissues were embedded in OCT (Bio Optica), frozen in dry ice, and stored at –80°C. Tissues were stained with the following primary antibodies: purified rat monoclonal anti–panendothelial cell antigen (550563, clone Meca32, BD Pharmingen), diluted 1:100; rabbit monoclonal anti–cleaved caspase-3 (asp175, clone 5A1, Cell Signaling Technology), diluted 1:100; rabbit monoclonal anti-CD34 (ab174720, Abcam) diluted 1:50; and sheep polyclonal anti-CLEC14A (AF4968, R&D Systems, using a batch that cross-reacted with mouse CLEC14A) diluted 1:50. Tissues were incubated with primary antibodies overnight at 4°C. After washing, samples were incubated for 1 hour at room temperature with the following secondary antibodies: anti–rat–Alexa Fluor 488 (A21208) or –Alexa Fluor 350 (A21093); anti–rabbit–Alexa Fluor 488 (A21206) or –Alexa Fluor 555 (A31572); and anti–sheep–Alexa Fluor 488 (A11015) (Thermo Fisher Scientific). Samples were then counterstained with DAPI Nucleic Acid Stain (Invitrogen). To detect CAR-transduced T cells, tissues were stained overnight at 4°C with rabbit monoclonal anti-CD34 (ab174720, Abcam; diluted 1:50 in PBS) and then washed and incubated for 1 hour at room temperature with anti–rabbit–Alexa Fluor 555 (A21429, Invitrogen) and counterstained with DAPI. Tissue from Rip-Tag2 and PDAC mice were analyzed using a Leica TCS SP2 AOBS confocal laser scanning microscope (Leica Microsystems). Acquisitions were performed with the same settings on multiple tissue sections and included negative controls for determination of background staining, which was negligible. Vessel density was quantified using ImageJ (NIH) software as the area occupied by Meca32^+^ structures compared with the total tissue area. For each animal, the total vessel area of at least 4 field/images was quantified. To analyze CLEC14A or cleaved caspase-3 expression, we identified vascular regions in the tissue from Meca32 staining and then determined the percentage of this area that was also positively stained for CLEC14A or cleaved caspase-3. All other tissues were analyzed using an Axiovert 100M laser scanning confocal microscope (Carl Zeiss).

Tissue sections from *Clec14a*-knockin mice (see below) were dewaxed in xylene and isopropanol. Endogenous peroxidase activity was blocked by 5 minutes of incubation in a 3% hydrogen peroxide, 90% methanol solution. High pH antigen unmasking solution (Vector Laboratories) was heated in an 850W microwave oven for 5 minutes; the tissue sections were placed into the solution and heated for a further 15 minutes before being left to cool for 10 minutes. Nonspecific antibody interactions were then blocked by 30 minutes of incubation with 10% horse serum in PBS. Tissues were then stained for human CLEC14A using a batch of polyclonal primary antibody AF4968 (1.7 μg/mL; R&D Systems) that did not cross-react with mouse CLEC14A, or concentration-matched isotype control (5-001-A; R&D Systems), for 1 hour at 23°C, followed by anti–sheep HRP (dilution 1:100; HAF016; R&D Systems) for 1 hour at 23°C. Immunohistochemical staining was developed using an ImmPACT NovaRED Peroxidase substrate kit (Vector Laboratories), incubating at 23°C for 3 minutes. Tissues were then counterstained with hematoxylin. Images were acquired using a Zeiss Axioskop 40 microscope.

### In vivo experiments

Mice were housed in individually ventilated containers with a 12-hour day/night light cycle at temperatures of 21°C ± 2°C and relative humidity of 55% ± 5%. All mice were allowed free access to water and a maintenance diet (Eurodent diet, 14%). All cages contained wood shavings, bedding material, and a plastic house.

#### Exploratory safety testing.

Six- to 8-week-old C57BL/6J mice (Charles River Laboratories) received nonmyeloablative (4 Gy) TBI. Eighteen hours later, each mouse received 2 × 10^7^ CAR- or mock-transduced T cell preparations from CD45.1^+^ congenic BoyJ mice (Charles River Laboratories) by tail vein injection. Mice were monitored for signs of toxicity, and immune monitoring was conducted by flow cytometric analysis of weekly tail bleeds. Mice were culled 45 days later, and major organs were removed for histological analysis.

#### Human Clec14a-knockin mouse.

C57BL/6J mice were engineered by Jackson Laboratories to express the human rather than mouse version of the extracellular domain of CLEC14A protein. A chimeric gene construct that encoded the human signal peptide and extracellular domain of CLEC14A and the mouse transmembrane and intracellular domains were inserted into the start codon of the endogenous mouse *Clec14a* gene, putting the latter out of frame and preventing transcription of the WT mouse *Clec14a* gene. Gene insertion was conducted using CRISPR/Cas9-based gene editing with the following guide RNAs: CLEC14A_up_sgRNA1, 5′-CAGCTGCATGTTGAGTCTCC-3′; CLEC14A_up_sgRNA2, 5′-CATGTTGAGTCTCCAGGATG-3′; CLEC14A_down_sgRNA1, 5′-GGCAAGCGCTGGCCTCATCC-3′; and CLEC14A_down_sgRNA2, 5′-AGGAGGCACAGGGCAAGCGC-3′.

The identity of the knockin mouse was confirmed by PCR genotyping.

#### Wound healing assay.

C57BL/6J mice were injected s.c. into the flank with 1 × 10^6^ LLC cells. Three days later, they were irradiated (4 Gy) and injected i.v. with 10 × 10^6^ CAR T cells (with 75% of cells expressing the CAR) or mock T cells (*n* = 7 mice per group). One day after T cell injection, skin wounds were administered with a 4 mm punch biopsy needle to the opposite flank of the mouse. Wound healing was assessed for the following 8 days, measuring the wound area with ImageJ from photographs taken alongside a scale.

#### Rip-Tag2 transgenic mouse tumor model.

Generation of RIP-Tag2 mice as a model of pancreatic islet cell carcinogenesis has been previously reported (53). Male RIP-Tag2 mice (provided by Doug Hanahan, ISREC, EPFL, Lausanne, Switzerland) were maintained on a C57BL/6J background (The Jackson Laboratory). From 12 weeks of age, all RIP-Tag2 mice received 50% sugar food (Harlan Teklad) to relieve hypoglycemia induced by the insulin-secreting tumors. At 12 weeks of age, mice received 4 Gy TBI using 6 MV x-ray produced by Tomotherapy Hi Art Unit (Accuray Inc.). Before treatment, a high-resolution CT scan virtual simulation (Toshiba Aquilion LB) was performed in order to define the treatment plan. Megavoltage CT verified the correct positioning of the mouse; then, TomoDirect Intensity Modulated Radiation Therapy was applied, allowing the delivery of 95% of the prescribed dose to the animal. The following day, cryopreserved CAR- and mock-transduced T cell preparations were thawed and washed, and 15 × 10^6^ T cells/mouse were delivered systemically by tail vein injection. Note the CAR T cell preparation contained 2.4 × 10^6^ T cells expressing CAR5.28z. Total tumor burden in culled CAR T cell–treated mice was quantified at 16 weeks of age using calipers to measure individually excised macroscopic tumors (>1 mm^3^) using the formula volume = a × b^2^ × 0.52, where a and b represent the longer and shorter diameter of the tumor, respectively. Volumes of all tumors from each mouse were added to give the total tumor burden per animal. There are no age-matched control comparisons for the 16-week CAR-treated mice, since untreated RIP-Tag2 mice do not survive to 16 weeks; thus, the comparison was made to 14-week-old mock-treated mice.

#### PDAC model.

The orthotopic mouse model of pancreatic ductal adenocarcinoma (mPDAC) has been described previously (50). Briefly, FVB/n syngeneic mice (Charles River Laboratories) were injected orthotopically in the pancreas with mPDAC cells (5 × 10^3^ cells/mouse in 50 μL of PBS). One week later, mice were conditioned by i.p. injection of cyclophosphamide (200 mg/kg). Twenty-four hours later, cryopreserved CAR-transduced T cells (of which 70% expressed CAR5.28z) and mock-transduced T cells were thawed and washed, and 20 × 10^6^ T cells/mouse were injected i.v. Three weeks after T cell infusion, mice were culled, and the pancreas was removed. Total tumor burden was quantified as described above.

#### LLC mouse model.

Six- to 8-week-old female C57BL/6J mice (Charles River Laboratories) were inoculated s.c. on the flank with 1 × 10^6^ LLC cells. Three days later, mice received 4 Gy TBI, and 18 hours after that, each mouse received 2 × 10^7^ CAR or mock T cell preparations from CD45.1^+^ congenic BoyJ mice by tail vein injection. CAR T cell preparations expressed either CAR3.28z or CAR5.28z with 11% and 7% of cells expressing the CAR, respectively. Tumor growth was measured with calipers, with bioluminescence imaging (IVIS Spectrum, Caliper Life Sciences), and by weight (after resection).

### Statistics

All statistical analyses were performed using GraphPad Prism software. Statistical tests used in this study were as follows: Wilcoxon matched-pairs signed rank test, unpaired 2-tailed t test, 2-tailed Mann-Whitney *U* test, and Kruskal-Wallis test. *P* < 0.05 was considered significant.

### Study approval

Studies with human donors were approved by the National Research Ethics Service Committee West Midlands, and all donors gave written informed consent before inclusion in the study. All procedures with Rip-Tag2 and mPDAC mouse models were approved by the Ethics Committee of the University of Turin and by the Italian Ministry of Health, in compliance with international laws and policies. All other mouse studies were performed under UK Home Office authorization.

## Author contributions

XZ, FM, JR, MB, BK, KW, JB, EJ, NJ, and SPL designed and performed experiments and analyzed the data. PG, E. Garibaldi, and ED helped with experimental design, as did DG, who also contributed essential reagents. Data analysis was also conducted by ZN, E. Giraudo, and RB. SPL and XZ wrote the manuscript, which was critically reviewed by FM, JR, E. Giraudo, and RB. SPL and RB conceived of and directed the research.

## Supplementary Material

Supplemental data

## Version 1 10/02/2020

Electronic publication
