# Supplementary material for: CAR T cells targeting tumor endothelial marker CLEC14A inhibit tumor growth
Source: JCI Insight. 2020 Oct 2;5(19):e138808. doi: 10.1172/jci.insight.138808 (PMC7566713; doi:10.1172/jci.insight.138808)
Supplement: Supplemental data [file jciinsight-5-138808-s009.pdf]

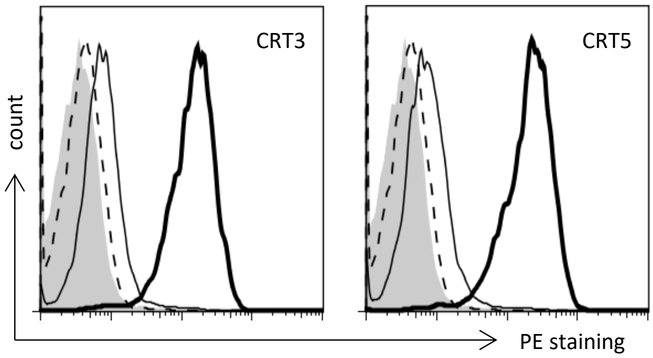

Supplementary Figure S1: Monoclonal antibodies demonstrate specific binding to CLEC14A

Flow cytometric analysis of HUVECs stained with monoclonal antibodies CRT3 and CRT5 where CLEC14A expression has been selectively inhibited by siRNA knockdown. Binding of these monoclonals was detected with a PE-conjugated anti-mouse secondary antibody. Heavy solid black line = CRT3 or CRT5 antibody staining of HUVECs transfected with control siRNA duplex. Solid black line = CRT3 or CRT5 antibody staining of HUVECs transfected with CLEC14a siRNA duplex. Dashed line = isotype control staining of HUVECs transfected with CLEC14a siRNA duplex. Grey shaded profile = isotype control staining of HUVECs transfected with control siRNA duplex.

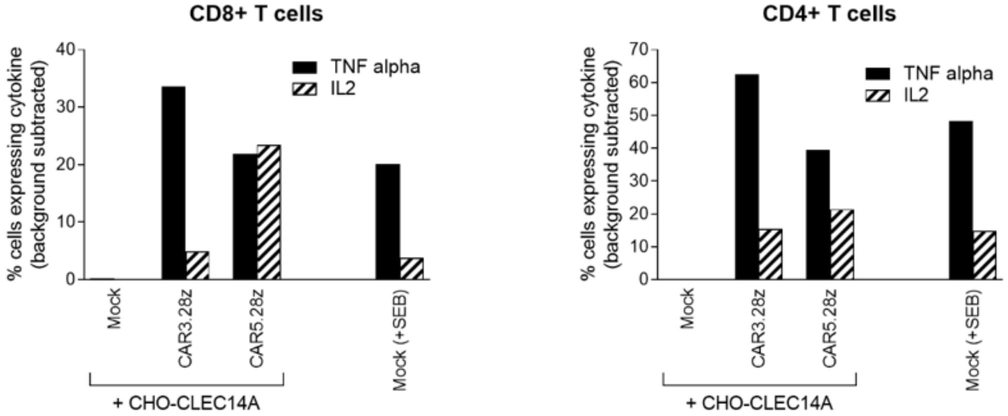

Supplementary Figure S2: TNFalpha and IL2 production by CAR T cells.
(A) Intracellular cytokine staining of TNF alpha and IL2 expression by CAR T cells when stimulated with CHO cells expressing CLEC14A. Data show percentage of CD8+ or CD4+ CAR T cells that expressed the cytokine having subtracted background responses to CHO cells transduced with the empty vector. Mock-transduced control T cells did not produce either cytokine when stimulated with CLEC14A but produced both when stimulated with staphylococcal enterotoxin B (positive control)

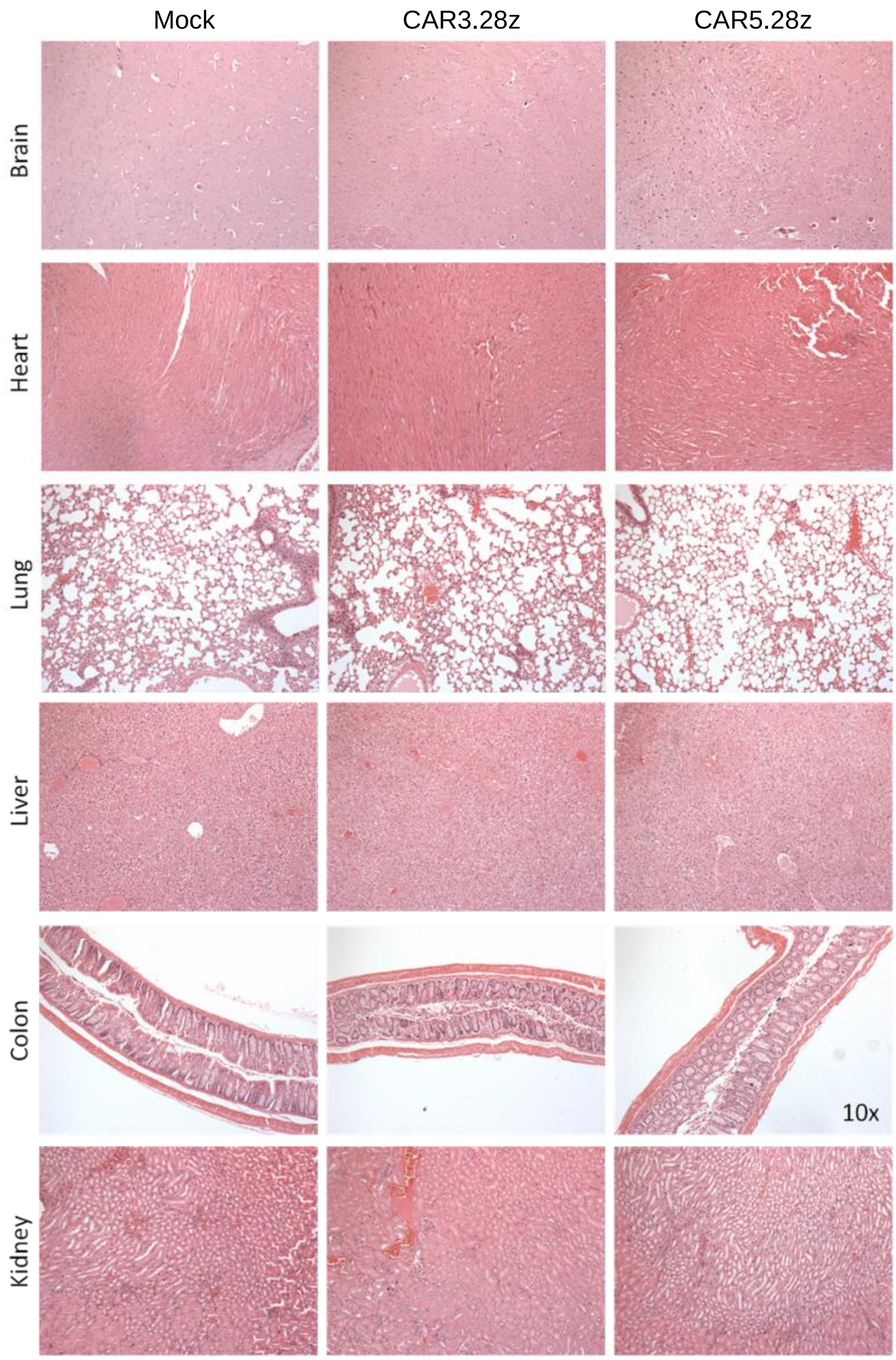

Supplementary figure S3: Histological analysis of tissues from mice treated with CLEC14A-specific CAR T-cells

Forty-five days after infusion of mouse T-cells expressing the CLEC14A-specific CARs CAR3.28z or CAR5.28z (or mock-transduced T-cells as a control), mice were sacrificed and vital organs collected and H&E stained for analysis.

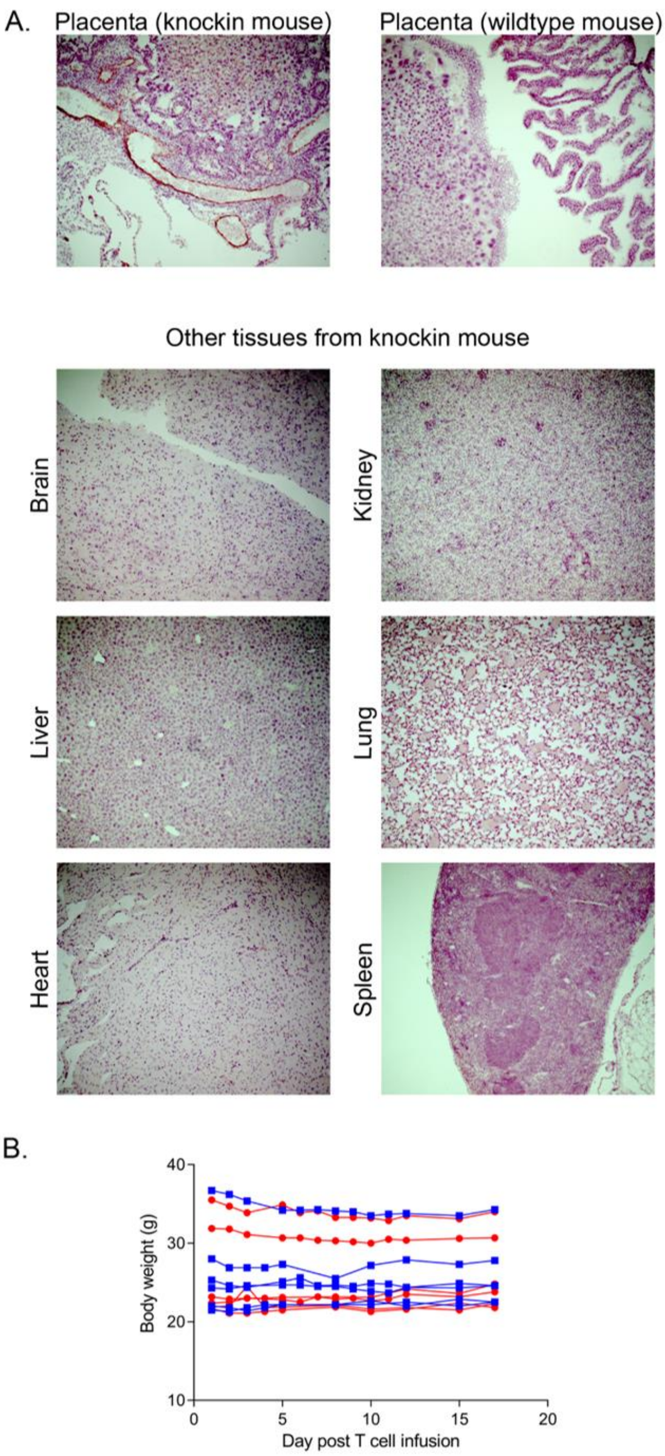

Supplementary Figure S4: Toxicity testing of CLEC14A-specific CAR T-cells in human CLEC14A knockin mice

(A) Representative images of healthy tissues from knockin mice expressing the human CLEC14A extracellular domain, stained for human CLEC14A expression. Placental tissue from a wildtype C57BL6 mouse is included as a negative control to demonstrate staining is specific for the human version of this protein. (B) Human CLEC14A knockin mice were irradiated (4Gy) on day 0 and 10 million CLEC14A-specific CAR- or Mock-transduced mouse T-cells infused on day 1. Mice displayed no signs of toxicity as illustrated by relatively stable body weights over time post infusion. Blue lines = CAR treated mice (n=6). Red lines = Mock treated mice (n=6). Independent histology review of major organs from these mice confirmed the lack of pathology.
